# Intellectual Property and Access to ART: Authors' Reply

**DOI:** 10.1371/journal.pmed.0030510

**Published:** 2006-11-28

**Authors:** Arachu Castro, Michael Westerhaus

**Affiliations:** Harvard Medical School, Boston, Massachusetts, United States of America; Brigham and Women's Hospital, Boston, Massachusetts, United States of America

We would like to thank Richard Stallman [1] for emphasizing the distinction between intellectual property law and patent law, which was not fully elucidated in our article (“How Do Intellectual Property Law and International Trade Agreements Affect Access to Antiretroviral Therapy?”) [2]. However, we don't feel that this distinction detracts from our overall argument that restrictions placed on medicines in the name of protecting “intellectual property” hurt efforts to expand access to essential medicines throughout the world. It is not so much the specific distinctions between patenting and intellectual property that interest us, but general acceptance of the principle that life-saving medicines can be “owned” and kept from those most afflicted by disease—often living in poverty—based on this concept of ownership.
